# Therapeutic application of extracellular vesicles in kidney disease: promises and challenges

**DOI:** 10.1111/jcmm.13407

**Published:** 2017-10-30

**Authors:** Lin‐Li Lv, Wei‐Jun Wu, Ye Feng, Zuo‐Lin Li, Tao‐Tao Tang, Bi‐Cheng Liu

**Affiliations:** ^1^ Institute of Nephrology Zhong Da Hospital Southeast University School of Medicine Nanjing China

**Keywords:** extracellular vesicle, exosome, kidney disease, therapy

## Abstract

Extracellular vesicles (EVs) are nanosized, membrane‐bound vesicles released from different cells. Recent studies have revealed that EVs may participate in renal tissue damage and regeneration through mediating inter‐nephron communication. Thus, the potential use of EVs as therapeutic vector has gained considerable interest. In this review, we will discuss the basic characteristics of EVs and its role in nephron cellular communication. Then, the application of EVs as therapeutic vector based on its natural content or as carriers of drug, in acute and chronic kidney injury, was discussed. Finally, perspectives and challenges of EVs in therapy of kidney disease were described.

**Table:** 

•**Introduction**
**•General characteristics of EVs**
‐ EVs biogenesis and release
‐ Mechanisms of EVs uptake by recipient cells
**•EVs in nephron cellular communication**
**•Therapeutic application of naturally secreted EVs in kidney disease**
‐ MSC‐EVs in therapy of acute kidney injury
‐ MSCs‐EVs in the treatment of chronic kidney injury
‐ Therapeutic role of other sources of cell‐derived EVs
‐ Mechanisms of therapeutic activity of naturally secreted EVs
**•EVs as therapeutic carriers in kidney disease**
‐ EVs loading with drugs for therapy
‐ EVs loading with nucleic acid for therapy
**•Target therapy with EVs in kidney disease**
**•Perspectives and challenge**
**•Acknowledgements**
**•Conflict of interest**

## Introduction

Extracellular vesicles (EVs) are nanosized, membrane‐bound vesicles released from different cells; recent studies have revealed that EVs play pivotal roles in both physiological intercellular crosstalk and disease pathogenesis 1. The content of EVs includes proteins, lipids, nucleic acids and membrane receptors loading from parent cells 2. EVs released into the extracellular space can enter body fluids and potentially reach distant tissues. Once taken up by neighbouring and/or distal cells, EVs transfer functional cargo that may alter the status of recipient cells, thereby contributing to both physiological and pathological processes 3. Thus, the potential use of EVs as therapeutic vector has gained considerable interest in different disease.

**Figure 1:**
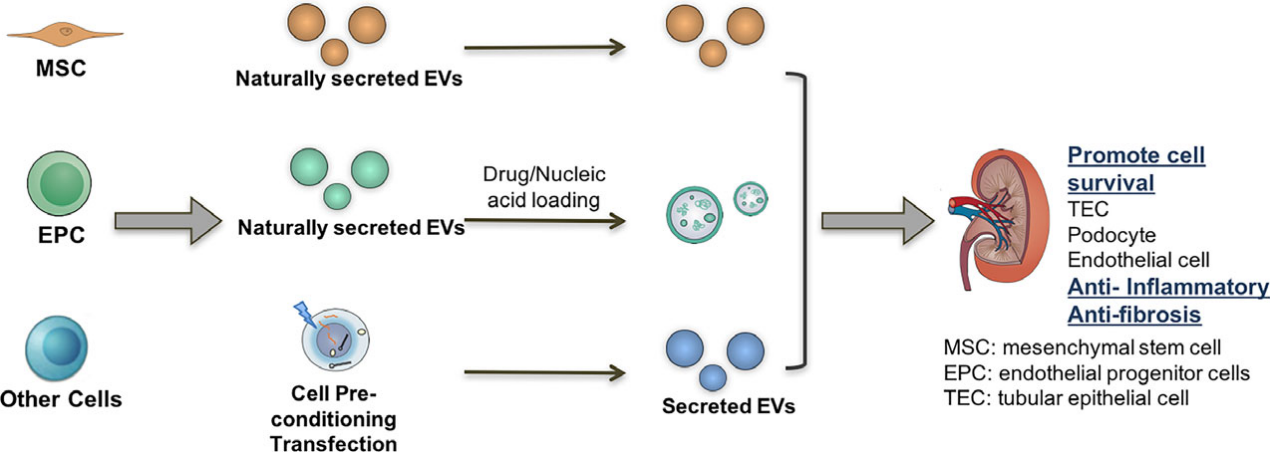
Application of extracellular vesicles (EVs) as therapeutic vector based on its natural content or as carriers of drug and nucleic acid in acute and chronic kidney injury is described in recent studies.

Traditionally, signalling of soluble cytokines and inflammatory mediators are considered the critical players in pathogenesis of kidney disease. It has been proposed that EVs also may participate in renal tissue damage and regeneration through mediating inter‐nephron communication 4, 5. In this review, we will focus on the therapeutic application of EVs in kidney disease. We will first discuss the basic characteristics of EVs and its role in nephron cellular communication. Then, the application of EVs as therapeutic vector based on its natural content or as carriers of drug and nucleic acid, in acute and chronic kidney injury, will be described (Table 1, Fig. 1). Finally, challenges in clinical translation of EVs‐based therapy are also discussed.

**Table 1:** Therapeutic application of extracellular vesicles in kidney disease

Cell source	EVs isolation	EVs doses	Injection method	Kidney injury model	Effective molecules	Naturally secreted (N)/manipulated (M)	References
BM‐MSCs	Ultracentrifugation	200 μg exosomes	Renal capsule injection	IRI	CCR2 protein	N	34
BM‐MSCs	Ultracentrifugation	2.2 × 10^8^ particles	Tail vein injection	Glycerol‐induced AKI	MicroRNAs	N	35
Umbilical cord MSCs	Ultracentrifugation	100 μg EVs	i.v. injection	IRI	MicroRNAs	N	36
Adipose tissue‐derived autologous MSCs	Ultracentrifugation	EVs (1 × 10^10^ particles)	Stenotic renal artery injection	Metabolic syndrome and renal artery stenosis	IL 10	N	37
BM‐MSCs	Ultracentrifugation	100 μg of MVs; 100 + 50 μg	i.v. injection	Cisplatin AKI	Whole content	N	39
Glomeruli MSCs	Ultracentrifugation	EVs produced overnight by 1 × 10^5^ cells	Tail vein injection	IRI	MicroRNAs	N	40
BM‐MSCs	Exosome precipitation solution, ExoQuick	1 × 10^6^ particles	i.v. injection	UUO	miRNA‐let7c	Plasmid transfection (M)	28
BM‐MSCs	Ultracentrifugation	30 mg MV	Caudal veins injection	UUO	microRNAs	N	43
Kidney‐derived MSCs	Ultracentrifugation	2 × 10^7^ particles	Tail vein injection	UUO	Whole content	N	44
Urine‐derived MSCs	Ultracentrifugation	100 μg exosome	Tail vein injection	Type I diabetes	VEGF, TGF‐β1, angiogenin and BMP‐7	N	48
ECFCs	Ultracentrifugation	20 μg exosome	Jugular vein injection	IRI	miR‐486‐5p	N	53
Endothelial progenitor cells	Ultracentrifugation	30 μg MVs	i.v. injection	IRI	miR‐126 miR‐296	N	29
Umbilical cord blood‐derived ECFCs	Ultracentrifugation	15 μg exosome	Jugular vein injection	IRI	Whole content	N	54
BM‐MSCs	Ultracentrifugation	30 μg MVs	Tail vein injection	UUO	microRNAs	Erythropoietin treatment (M)	83

IRI, ischaemia–reperfusion injury; AKI, acute kidney injury; UUO, unilateral ureteral obstruction; MSCs, mesenchymal stem/stromal cells; ECFCs, endothelial colony‐forming cells; EVs, extracellular vesicles; BM, bone marrow; MV, microvesicle.

## General characteristics of EVs

### EVs biogenesis and release

EVs encompass a vast heterogeneous population of membrane‐bound vesicles, which are classified as exosomes, microvesicles and apoptotic bodies based on their size and biogenesis in most reviews and have been extensively discussed elsewhere 6. EVs biogenesis and its content are not only determined by their cellular source but are also sensitive to cellular status and environmental changes. Increased exosome release has been reported in hypoxia, acidic pH, heat shock and oxidative stress conditions 7.

In kidney, EVs secretion was also regulated by microenvironmental change. EVs secreted by multiple types of kidney intrinsic cells have been studied, including endothelial cells, podocyte and epithelial cells facing urinary tract. The increasing evidence indicated that the EVs biogenesis and the packaging content are influenced by pathologic conditions, like high glucose, pro‐fibrotic cytokines 8, 9, 10, 11. Urinary EVs excretion and its C‐megalin content increased along with the progression of the albuminuric stages in T2DM patients. When rat proximal tubule cells were treated with advanced glycation end product‐modified BSA, it caused apparent lysosomal dysfunction, which stimulated multivesicular body formation, resulting in increased exosomal C‐megalin excretion 12. In hypertensive patients, urinary podocyte‐derived EVs levels are elevated in patients with renovascular hypertension and low eGFR compared with patients with essential hypertension and relatively preserved renal function 8. Moreover, increased plasma level of vasopressin promoted the excretion of urinary exosomal AQP2 7. However, the underlie mechanism of EVs biogenesis and release in kidney is not clear yet.

### Mechanisms of EVs uptake by recipient cells

The application of EVs in therapy of disease depends upon efficient EVs uptake by recipient cells and the transfer of active molecules. Recipient cells can uptake vesicles *via* direct fusion of EVs and recipient cell plasma membrane through endocytosis, macropinocytosis and phagocytosis 13, 14, 15, 16.

The uptake of EVs to recipient cells was regulated or controlled by critical molecules and microenvironment conditions. Oosthuyzen *et al*. recently reported the potential hormonal regulation of EVs transfer. Desmopressin, a vasopressin analogue, stimulated the uptake of fluorescently loaded ECVs into kidney collecting duct cell 17. Besides, microenvironmental pH is a key factor for exosome traffic. An increased exosome release and uptake by melanoma cells at low pH occurred when compared with a buffered condition 13. Exosome internalization could be inhibited by the knockdown of dynamin 2 or overexpression of a dominant‐negative form of dynamin 2. Antibody pretreatment assays demonstrated that tim4 but not tim1 was involved in exosomes uptake 15. Interestingly, exosome adhesions were directed to specific organ and cell through integrins on exosome. Ayuko *et al*. found that integrins expressed on tumour‐derived exosomes dictate exosome adhesion to specific cell types and extracellular matrix molecules in particular organs, which determined future metastatic sites 18.

## EVs in nephron cellular communication

An increasing body of evidence indicates that EVs play a pivotal role in cell‐to‐cell communication. EVs may directly stimulate target cells by receptor‐mediated interactions or may transfer bioactive molecules including membrane receptors, proteins, mRNAs, microRNAs and organelles 5. EVs may mediate the cellular communication across different regions of nephron, including crosstalk between glomerular cells, cells between glomerular and tubules and signal transfer between different segments of tubules 19.

Urinary EVs are released into urine from all regions of the nephron and were readily identified as markers specific to the cell of origin. Miranda *et al*. were able to detect mRNA in the human urinary microvesicles encoding proteins from all regions of the nephron and the collecting duct 20. *In vitro* study has shown that exosome content may transfer from renal proximal tubule cells to human distal tubule and collecting duct cells 21. It is reasonable to speculate that release and downstream reuptake of urinary EV contents could affect the function of the recipient cell 6, 22. Interestingly, normal human urinary exosomes are significantly enriched for innate immune proteins. It may act as antibacterial immune effectors by inhibiting the growth of pathogenic and commensal *Escherichia coli* and inducing bacterial lysis 23. Besides, injured tubular cells pass exosomes containing TGF‐β1 to fibroblasts, which may represent a new crosstalk between the tubular epithelium and the interstitial fibroblast that mediates progression of the disease 24. Tubular cells under transforming growth factor‐β1 treatment can secrete miR‐21 and deliver it into recipient tubules by EVs, where the exogenous miR‐21 can target PTEN protein and enhance Akt signalling in recipient cells 11.

Thus, vesicles from injured cells may accelerate inflammation and fibrosis, whereas those from cells with regenerative potential appear to promote cell survival 25. This highlights the possibility by targeting EVs as novel therapeutic approach for kidney disease.

## Therapeutic application of naturally secreted EVs in kidney disease

Studies have shown that EVs from various cell sources have therapeutic effect through its intrinsic contents, which include activated antigen‐presenting cells (APC) 26, natural killer cells (NK) 27, mesenchymal stem cell (MSC) 28 and endothelial progenitor cell (EPCs) 29. Besides, tumour‐derived EVs contain and transfer tumour antigens to APC, promoting antitumour effects 30. In kidney disease, the most studied naturally secreted EVs for therapeutic purpose are from mesenchymal stem/stromal cells (MSCs) and endothelial progenitors.

### MSC‐EVs in therapy of acute kidney injury

**Figure 2:**
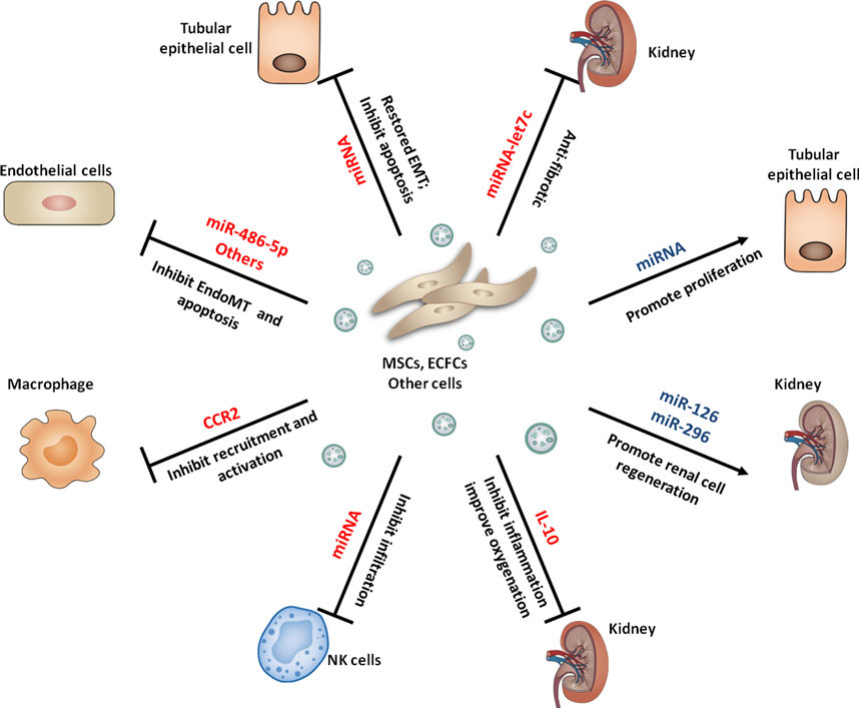
Target cell and effective molecules (miRNA) in EVs‐based therapy of kidney injury are schematically summarized. EVs: extracellular vesicles; MSCs: mesenchymal stem cells; ECFCs: endothelial colony‐forming cells; EndoMT: endothelial‐to‐mesenchymal transition; EMT: epithelial–mesenchymal transition.

Mesenchymal stem/stromal cells have distinct capability for renal repair. Interestingly, MSC‐derived EVs recapitulate immunomodulatory and cytoprotective activities of their parent cells 31. Increasing evidence has shown that delivery of EVs derived from MSCs could restore renal structure and function in different experimental models of acute kidney injury (AKI). MSC‐EVs may protect kidney from injury through their properties of immunomodulatory, anti‐apoptosis and proliferation stimulation. The target cell and effective molecules (miRNA) in EVs‐based therapy of kidney injury are schematically summarized in Figure 2.

At the immunobiological level, MSC‐EVs treatment mainly led to a reduction of pro‐inflammatory and an increase of anti‐inflammatory cytokines. Macrophages and lymphocytes were suppressed consequently. In a variety of *in vivo* models, it has been observed that MSC‐EVs suppress pro‐inflammatory processes and exert kidney repair effect.

We and other researchers have demonstrated that activation and infiltration of macrophage play an important role in the development of AKI. Therefore, reducing infiltration and activation of macrophage may be one of the directions of AKI treatment 32, 33. Interestingly, Shen *et al*. have recently documented that CCR2 high‐expressed MSC‐exo could reduce the concentration of free CCL2 and suppress its functions to recruit or activate macrophage in kidney. And CCR2 knockdown impaired the protective effects of MSC‐exo for the renal ischaemia/reperfusion injury in mouse 34. It suggests that receptor proteins expressed on exosomes could also contribute to the therapeutic effect of MSC‐exo. Collino *et al*. reported that in glycerol‐induced AKI, down‐regulated genes related to fatty acid metabolism and up‐regulated genes related to inflammation, matrix‐receptor interaction and cell adhesion molecules were observed. These alterations reverted after treatment with MSC‐EVs 35. Besides, MSC‐EVs could also ameliorate renal ischaemic reperfusion injury by decreasing NK cells. The labelled MSC‐EVs were found in ischaemic kidneys, spleen and lungs at the 24 hrs after injection 36.

Interleukin (IL)‐10 is an anti‐inflammatory cytokine that regulates the functions of immune cells and a key determinant of alternatively activated (M2) macrophage phenotype, which is well known to attenuate inflammation and promote tissue repair. Studies have demonstrated that IL10 may mediate the repair activity of MSC‐EVs. Eirin *et al*. tested whether MSC‐EVs attenuate renal inflammation in model of metabolic syndrome and renal artery stenosis. Abdominal subcutaneous adipose tissue (5–10 g) was collected and processed for MSC isolation. EVs were obtained from supernatants of 10 × 10^6^ MSCs. And after four weeks of intrarenal injection, EVs fragments colocalized with stenotic‐kidney tubular cells and macrophages, indicating efficient delivery of EVs to kidney. They found EVs delivery attenuated renal inflammation and improved medullary oxygenation and fibrosis through their cargo of cytokine IL10 37. Ragni *et al*. demonstrated that IL10 mRNA was selectively enriched in MSC‐EVs and could be acquired by tubular cells, which was efficiently translated into protein. MSC‐EVs enriched with IL10 mRNA protect tubular cells from acute injury by cisplatin treatment 38.

Besides, cultured human TECs with MSCs‐EVs significantly inhibited apoptosis induced by cisplatin, which suggested that the mechanism of protection was mainly ascribed to an anti‐apoptotic effect of MVs 39. Interestingly, EVs originated from resident population of MSCs within the glomeruli (Gl‐MSCs) promoted kidney recovery of AKI induced by ischaemia–reperfusion injury. Ranghino *et al*. reported that Gl‐MSCs and Gl‐MSC‐EVs both ameliorate kidney function and reduce the ischaemic damage by enhancing tubular epithelial cell proliferation 40.

### MSCs‐EVs in the treatment of chronic kidney injury

Although most of the recent studies focused on the therapeutic role of MSC‐EVs in AKI animal model, some reports tried to explore its effects on chronic kidney injury model. The EVs that have been studied were originated from MSCs cultured from bone marrow, kidney glomerular or urine. Bruno *et al*. reported that in cisplatin‐induced kidney injury model, the single administration of MSCs‐MVs ameliorated renal function and morphology, and improved survival but did not prevent chronic tubular injury and persistent deterioration of renal function. Interestingly, multiple injections of MVs further decreased mortality, and at day 21, surviving mice showed normal histology and renal function 39.

Renal fibrosis is the key cellular process in the progression of CKD and thereby represents an excellent treatment target. Despite a number of potential anti‐fibrotic treatment targets like TGF‐β and CTGF, identified in preclinical studies, translation to clinical trials has remained insufficient 41. Recent studies showed that EVs are not only active players in promoting renal fibrosis 24 but also might be potential therapy vector for CKD by ameliorating renal fibrosis. Gallo *et al*. demonstrated that stem cell‐derived EVs were able to interfere with mesangial cells (MCs) collagen production in a hyperglycaemic (HG) setting. Both MSC and human liver‐derived EVs protect MCs from HG‐induced TGF‐β expression and matrix protein synthesis *via* the transfer of miR‐222 42. Wang *et al*. showed that in mice with unilateral ureteral obstruction (UUO), MSCs engineered to overexpress miRNA‐let7c could attenuate kidney injury and significantly down‐regulate collagen IVα1, metalloproteinase‐9, transforming growth factor (TGF)‐β1 and TGF‐β type 1 receptor (TGF‐βR1) in UUO kidneys, compared with controls. The transfer of miR‐let7c from MSCs occurred *via* secreted exosome 28. Another study showed that the EVs from non‐modified MSC reversed deterioration of kidney function in UUO model, with superior therapy effect than MSC 43. Anti‐fibrotic effect of EVs from kidney‐derived mesenchymal stem cells was observed by ameliorating endothelial‐to‐mesenchymal transition (EndoMT) and improved peritubular capillaries rarefaction in UUO kidneys 44.

Diabetic nephropathy (DN) is a common cause of end‐stage renal disease worldwide. MSC‐based therapy may bring substantial benefit for patients suffering from DN based on its pleiotropic properties of immunomodulation, inhibition of apoptosis, fibrosis, oxidative stress, etc. 45. Recent studies found that EVs and its packaged molecules might be useful biomarker for DN in clinical patients 46, 47. More importantly, exosomes from conditioned medium of urine‐derived stem cells (USCs‐Exo) could prevent DN, by inhibiting podocyte apoptosis and promoting vascular regeneration and cell survival 48. MSC‐EVs could effectively prevent the onset of disease in type 1 diabetes by inhibiting activation of APC and suppress the development of T helper 1 (Th1) and Th17 cells 49. However, in‐depth studies are needed regarding the therapy potential of MSC, especially MSC‐EVs in DN.

Interestingly, a recent study investigated the therapeutic role of MSC‐EVs in CKD patients. Forty CKD patients (stage III and IV) (eGFR 15–60 mg/ml) have been divided into two groups: MSC‐EVs treatment group and matching placebo group. Results showed that MSC‐EVs are safe and can ameliorate the kidney inflammation and improve the overall kidney function in CKD patients 50. However, some other studies have reported that MSC therapy promoted renal fibrosis and failed to improve renal function in CKD animal model and clinical patients 51, 52. Thus, the role of MSCs in CKD is controversial, which suggests that the safety and efficiency of MSC‐EVs should be studied further. Although MSCs‐EVs have been found to promote comparable therapeutic activities as MSCs themselves, the different effects of MSCs and MSCs‐EVs on kidney repair need further investigation.

### Therapeutic role of other sources of cell‐derived EVs

Since EPCs showed a high proliferative capacity and pro‐angiogenic activity, EPCs have been explored as possible EVs source for kidney regeneration.

Endothelial colony‐forming cells (ECFCs) are early lineage EPCs. Several previous studies have demonstrated that ECFC‐derived exosomes may have therapeutic ability in AKI mice models. Vinas *et al*. reported that delivery of ECFC exosomes could reduce ischaemic kidney injury *via* transfer of miR‐486‐5p targeting PTEN 53. Cantaluppi *et al*. found that EVs from EPCs can be efficiently delivered to both renal capillaries and renal tubules. The EVs are rich in microRNA‐126 and microRNA‐296, which are partly contributed to EV protection to ischaemic AKI 29. In cultured human umbilical vein endothelial cells, ECFC exosomes inhibit apoptosis stimulated with hypoxia/reoxygenation. Besides, *in vivo* study in ischaemic AKI model showed that the protective effects of ECFC administration were associated with significant reductions in post‐ischaemic tubular necrosis and reductions in renal apoptosis 54.

Interestingly, EVs produced by human renal proximal tubular epithelial cells (RPTECs) may induce mesenchymal–epithelial transition of bone marrow‐derived MSCs. miR‐200 carrying EVs released from RPTECs that induced the epithelial commitment of MSCs may contribute to regenerative potential of MSCs 55. Thus, pretreatment of MSCs with EVs released from intrinsic kidney cells may represent a novel approach for accelerating the therapeutic effect of MSCs.

### Mechanisms of therapeutic activity of naturally secreted EVs

Mechanistically, most studies attributed the protective effect of MSC‐EVs on kidney diseases mostly to their RNA content, including mRNA and microRNAs. Collino *et al*. reported that EVs derived from the MSCs with silenced Drosha, a key enzyme for microRNA biogenesis, were ineffective in promoting recovery of glycerol‐induced AKI 35. RNase abolished the effects of microvesicles *in vitro* and *in vivo*, suggesting RNA‐dependent biological effects on repair of kidney injury 56. Further study showed that microvesicles shuttle a specific subset of cellular mRNA, which may activate a proliferative programme in surviving tubular cells 56. Overall, the horizontal RNA transfer may represent a major mechanism for MSC‐EVs restoring ability observed *in vivo* 38.

Extensive proteomic analysis of MSC‐EVs showed that the proteins related to cell proliferation, adhesion, migration and morphogenesis were present in the vesicles. The MSC‐EVs proteome provides a basis for understanding the mechanism of MSC‐EVs activity in tissue repair and regeneration 57. In addition, EVs derived from urine MSCs contained the potential factors, including growth factor, transforming growth factor‐β1, angiogenin and bone morphogenetic protein‐7, which may contribute to vascular regeneration and cell survival 48.

Collino *et al*. conducted a study that evaluated the functional properties on renal tubular cells of MSC‐EVs sub‐populations separated by gradient floatation. EVs were separated into low (CF1), medium (CF2) and high (CF3) floatation density fractions. Although renal cells internalized EVs derived from all fractions, CF2 fraction enriched in exosomal markers was the most active on stimulating cell proliferation and inhibiting apoptosis 58. Similarly, Burger *et al*. found that ECFCs exosomes inhibit apoptosis of endothelial cells stimulated with hypoxia/reoxygenation, but not microparticles (100–1000 nm diameter) or vesicle‐depleted conditioned medium. Consequently, exosome may represent the major effective subpopulation with protective effects in ischaemic AKI 54.

## EVs as therapeutic carriers in kidney disease

EVs have been shown to be superior to other existing delivery systems owing to its nanosized vesicles, high permeability, less immunogenicity and non‐cytotoxicity. Furthermore, exosomes may evade fast clearance by the mononuclear phagocyte system owning to its nanosized characteristic 59. EVs and incorporated RNAs showed high stability irrespective of certain times of freeze‐thaw cycles and long‐term storage 60. Thus, EVs provide an attractive approach to develop novel therapeutic carrier for disease treatment. Studies to date have explored small RNAs, mRNAs, proteins, drugs and small molecules as therapeutic cargo to be loaded in EVs. Briefly, there are two categories of therapeutic EVs loading approach: extracellular EV loading and intracellular loading. Intracellular approach is loading during biogenesis, which is purifying EVs directly from natural or transfection donor cells. Extracellular approach refers to depositing cargo into purified EVs through electroporation saponin permeabilization and hypotonic dialysis 31, 61.

After systemic administration, when the fate of delivered EVs *in vivo* was assessed using fluorescence labelling, most studies reported that unmodified exosomes accumulated preferentially in liver, kidney and spleen 62. It indicated that EVs might offer appealing therapeutic carrier for kidney disease.

### EVs loading with drugs for therapy

Although synthetic drug vehicles have successfully circumvented many problems, EVs are naturally occurring nanosized vesicles that have attracted considerable interests as drug delivery vehicles in the past few years 63. Of the cell types known to produce exosomes, MSC is the most well suited for mass production of exosomes for drug delivery 64.

Sun *et al*. loaded EVs with curcumin, an anti‐inflammatory drug, by mixing curcumin and exosomes to allow non‐specific binding. Intraperitoneal injection of curcumin‐loaded exosomes resulted in protection against lipopolysaccharide‐induced septic shock in mice 65. Pascucci *et al*. showed successful loading of paclitaxel (PTX) into EVs, by incubating MSCs with a high dosage of PTX, which were able to inhibit *in vitro* tumour growth 66. Haney *et al*. developed a new exosomal‐based delivery system for a potent antioxidant, catalase, to treat Parkinson's disease. They found that a reformation of exosomes upon sonication and extrusion, or permeabilization with saponin, resulted in high loading efficiency, sustained release and catalase preservation against protease degradation 67. However, the application of EVs as drug carrier remained to be explored.

### EVs loading with nucleic acid for therapy

miRNA have attracted increasing attention as critical regulators of various diseases through post‐transcriptional regulation of gene expression. Thus, interest has been spurred in developing therapeutic approach based on miRNA. The majority of therapeutic studies investigating miRNAs have so far focused on cancer; the miRNA overexpression, miRNA mimics or miRNA antagonist have been investigated for the therapeutic purpose of disease.

Engineered anti‐miRNA oligonucleotides can direct against specific miRNAs that bind specifically to individual miRNAs by sequence complementarity and block the specific miRNA function 68. The greatest concentration of anti‐miR oligonucleotides is found in the kidney and liver following systemic delivery. In the kidney, the proximal tubule concentrates on the oligonucleotides most strongly, indicating that anti‐oligonucleotide therapy may be effective for kidney disease 69. In a mouse model of Alport nephropathy, weekly delivery of anti‐miR21 oligonucleotides to diseased mice dramatically improved survival of Alport mice and reduced histological injury 70. A locked nucleic acid (LNA)‐modified inhibitor of miR‐192 significantly decreased renal fibrosis and improved proteinuria 71.

miRNA mimics are dsRNA molecules which separate to ssRNA in cell, and one strand loads into the RISC to function as a miRNA. However, miRNA mimic technology lags behind anti‐miRNA approaches in the therapy of kidney disease. Although miRNA mimics have been widely used in cultured cells, their application as drug candidates has been limited by delivery, triggering of TLR responses *in vivo* 69.

However, naked miRNA oligonucleotides are rapidly degraded in biofluids. Although LNA‐modified oligos 72 or modification with the phosphate backbone and sugar moieties of the anti‐oligonucleotide have made it more stable *in vivo* 70, the efficient delivery and stability need further improvement. Synthetic polymer and lipids as well as virus‐based vectors are among the most widely explored vehicles for miRNA delivery; however, it is proved unsatisfactory due to safety concerns, immunogenicity and low efficiency *in vivo* 73. EVs may display better biocompatibility and higher delivery efficiency for miRNA‐based therapy 74. Katakowski *et al*. transfect MSCs with a miR‐146b expression plasmid and harvested EVs released by the MSCs. Intra‐tumour injection of exosomes purified from miR‐146‐expressing MSCs remarkably reduced tumour growth in a rat model of primary brain tumour 75. MSCs were engineered to overexpress miRNA‐let7c, which was administered to mice of UUO model. Results showed that the therapy attenuated kidney injury *via* exosome delivery of exogenous miRNA‐let7c 28.

## Target therapy with EVs in kidney disease

Extracellular vesicles were systemically delivered in most studies, which reduced the therapeutic efficiency and may increase side effects in the treatment of disease. Thus, successful and efficient delivery of EVs to the site of injury remains a significant challenge in the field. To evaluate the therapeutic efficiency of MSC‐EVs, it is essential to investigate *in vivo* their biodistribution and localization within the injured kidneys. Grange *et al*. demonstrated that it was possible to analyse the biodistribution of EVs either by direct labelling or by the production of labelled EVs from MSCs. Five hours after injection intravenously (i.v.), EVs could be imaged *in vivo*. In particular, labelled MSC‐derived EVs were found to localize within the injured kidney 76. However, the biodistribution of EVs *in vivo* is affected by different EV doses, routes of injection and cellular origin of EVs, which should be considered in the study of EVs‐based therapy 77.

Targeting exosomes to a given cell type can be obtained by displaying a ligand on the outer surface of exosomes. It can be accompanied by inserting the coding sequence of the ligand to the coding sequences between the signal peptide and N‐terminus of the mature peptide of a transmembrane protein 78. The transmembrane proteins that have been used usually are LAMP‐2b, lactadherin and platelet‐derived growth factor receptors. Alternatively, EVs surfaces can be coated with antibody fragments recognizing target antigens on the specific target cells 79. Moreover, external magnetic field might be useful for targeting EVs to diseased organ or tissue. Qi *et al*. developed a dual‐functional exosome‐based superparamagnetic nanoparticle; the drug‐loaded exosome‐based vehicle enhanced cancer targeting under an external magnetic field and suppressed tumour growth 80.

Since studies demonstrated that GE11 peptide (amino acid sequence YHWYGYTPQNVI) binds specifically to EGFR, but is markedly less mitogenic than EGF, Ohno *et al*. investigated which ligand is better for targeting exosomes to EGFR‐expressing tumours. Modified exosomes with the GE11 peptide or EGF on their surfaces were intravenously injected to mice. Results showed that exosomes targeting EGFR delivered let‐7a specifically to xenograft breast cancer cells, and GE11 peptide is likely superior to EGF for targeting EGFR‐expressing tumours 81. Zhong *et al*. used ultrasound‐mediated gene transfer of inducible miR‐21 knockdown (miR‐21 KD) plasmids into the kidneys of mice. The mixture of miR‐21 KD plasmid in microbubbles combined with ultrasound transducer was applied to kidneys, targeting plasmid to kidney 82. However, the possibility of targeting exosome to kidney or specific kidney cell remained unclear. Coating antibodies specific to podocytes, tubular epithelial cells to EVs may be promising directions in future study. The feasibility of applying magnetic field and ultrasound in directing EVs in kidney may also be worth of investigation.

## Perspectives and challenge

Despite the therapeutic potential of EVs, there are still challenges in regard to the production, isolation and quantification of EVs for therapy purpose. The composition and amount of EVs released appear to differ depending on the parent cell type and the microenvironment. Therefore, the appropriate selection of donor cells and culture condition for exosome production is a key factor for clinical applications of EVs‐based therapies. Interestingly, EVs derived from MSCs incubated with erythropoietin (EPO) show superior protective effect in renal injury of UUO *in vivo*. The changed miRNA in EPO‐MVs may have contributed to their enhanced protective effects following renal injury compared to MSC‐MVs 83.

Besides, a large number of cells must be cultured *in vitro* in order to get a few micrograms of EVs, which limits the potential of EVs as a therapeutic carrier. Production of EVs can be enhanced by increasing intracellular calcium levels, thermal stimulation and hypoxia and changing the microenvironment pH 13, 84, 85, 86. Hollow‐fibre bioreactor was reported to be able to increase EVs production. The bioreactor culture yielded about 40‐fold more EV per mL conditioned medium, as compared to conventional cell culture 84. However, the development of novel strategies to enhance EVs production and recovery needs further improvement and validation 81.

Traditionally, EVs are isolated and purified by differential centrifugation and ultracentrifugation. Ultracentrifugation can produce homogeneous‐sized and intact vesicles 60. However, traditional approach is time‐consuming, and EVs production yield is not satisfying. In recent years, new isolation and purification method has been reported. Size‐exclusion chromatography successfully isolated EVs from umbilical cord MSC medium, and the well‐purified and defined preparations of MSC‐derived EVs may help to achieve the immunosuppressive effect for therapy 87. Similarly, a recent study found that size distribution could profoundly affect exosomes therapeutic potential. Polymer‐based precipitation leads to smaller particle size distributions, faster uptake by target cells and increased cellular motility 88.

In the clinical treatment context, the technical challenge of EVs quantification is a critical issue. While great advancement has been made to measure both EVs particle numbers and protein content 89, there is still an urgent need to standardize EVs quantification across laboratories. Currently, there is debate as to whether EVs should be quantified by number of vesicle particles, amount of vesicle protein or vesicle number to protein ratio. Recently, International Society for Extracellular Vesicles and ‘EV‐TRACK’ have provided researchers with a minimal set of biochemical, biophysical and functional standards that should be considered in EVs research 90, 91. The authors provided minimal standards for general characterization of EVs, characterization of single vesicles and recommendations for controls in studies of the functional activity of EVs. The recommendation should be useful to promote the consensus standards in the future study.

To define EVs dosage for clinical therapeutic purposes, the problem as to quantification of EV should be resolved and standardized as soon as possible 92.

Therefore, EVs have shown therapeutic potential in promoting kidney repair in both acute and chronic kidney injury. However, there is a continued need to address practical issues that deal with the manufacturing, purification and target administration of therapeutic EVs into the kidney. In addition, involvement of EVs in pathophysiological processes of kidney disease needs further investigation before conducting studies aimed to progress to clinic.

## Conflict of interest

None.
